# A Clustering Protocol for Wireless Sensor Networks Based on Energy Potential Field

**DOI:** 10.1155/2013/829861

**Published:** 2013-10-21

**Authors:** Zuo Chen, Yao Xiao, Xiaodong Li, Renfa Li

**Affiliations:** ^1^College of Information Science and Technology, Hunan University, Changsha, Hunan 410082, China; ^2^College of Environmental Science and Engineering, Hunan University, Changsha, Hunan 410082, China

## Abstract

It is the core issue of researching that how to prolong the lifetime of wireless sensor network. The purpose of this paper is to illustrate a clustering protocol LEACH-PF, which is a multihop routing algorithm with energy potential field of divided clusters. In LEACH-PF, the network is divided into a number of subnetworks and each subnetwork has a cluster head. These clusters construct an intercluster routing tree according to the potential difference of different equipotential fields. The other member nodes of the subnetworks communicate with their cluster head directly, so as to complete regional coverage. The results of simulation show that LEACH-PF can reduce energy consumption of the network effectively and prolong the network lifetime.

## 1. Introduction

Due to the increasing development of microelectromechanical system (MEMS), system on chip (SOC), and embedded technology, the wireless sensor network has sprung up. Wireless sensor network consists of numerous nodes integrated with microsensors, microprocessors, and microcommunication modules constructed in self-organizing way. It is widely used in perceiving the environmental temperature, pressure, and relative humidity, because of its low cost, huge amount, and intensive distribution [1–5].

In practical applications, wireless sensor networks are generally deployed in severe environments, which not only require the detective capability of the nodes, but also require the system to work effectively for a long time [6]. Since the nodes of WSN are very tiny, they will lead to a series of restrictions, such as limited computing power, transmission distance, limited amount of data, and limited node energy. The most prominent one of these problems is energy restriction. Due to the characteristic of tiny size, large number of nodes, and poor environment for deployment, it is almost impossible to replace battery manually. On the other hand, solar and other new sources of energy are inappropriate to large-scale applications for considering technology and cost. Therefore, in order to avoid reduction on the functionality of WSN, applying efficient routing algorithm is one of the most important methods to save system power and extend network lifetime.

Currently, the study of routing protocols for WSN topology control could be divided into three main issues: the plane routing protocol, the hierarchical routing protocol [7], and the location-based information routing protocol. In the plane routing protocol, all nodes have equal status. Their network structure is simple, robust, and easy to realize. However, its drawbacks are obvious, such as the lack of management node which makes it hard to optimize network resources and slow response to network dynamic changes. Thus, this kind of protocol is not suitable for the large-scale wireless sensor network. In the location-based information routing protocol, nodes need to reserve their neighbor's locating information. The hierarchical routing protocol divides nodes into different clusters, each of which consists of cluster head and cluster members. The cluster members gather information and form a simple many-to-one communication which does not need redundant routing information, and data are fused at the cluster head. That means this protocol can reduce energy consumption and has good expansibility.

## 2. Related Researches

The primary task of wireless sensor network is data collection and transmission. There are two kinds of wireless communication model: free space model and multipath fading model. There is a nonlinear relationship between each node in terms of the energy consumption of wireless communication. The energy consumption of free space model is much smaller than that of multipath fading model. In practical environment, there is only one sink node in a few hundred meters radius. When a node is far away from sink node, it may be run out of energy shortly, which would lead to quality reduction of network coverage.

The proposal of hierarchical routing effectively solved the problem of excessive energy consumption in the course of direct communication between node and sink node. The hierarchical routing intends to divide the whole wireless sensor network into various LANs. Then agent nodes are selected in LAN which are called cluster heads. They are responsible for the communication between LAN and sink node. Nodes in LAN communicate with the cluster head nodes by means of many-to-one, which will greatly shorten the communication distance between nodes and sink node and reduce the energy consumption of single node.

As a typical representative of the hierarchical routing protocol, (low energy adaptive clustering Hierarchy) LEACH [8], proposed by Heinzelman, and so forth, is a low-power adaptive clustering routing protocol.

The shortcomings of LEACH are as follows.

1. The probability of becoming a cluster head is the same to each node. Once the low energy node becomes a cluster head node frequently, it would run out its energy fast and make the network incomplete.
2. There is uneven distribution of cluster head in each region. Some regions may have more cluster heads relatively, because the cluster head is randomly generated.
3. Cluster heads communicate with sink node by single-hop routing. Some cluster heads which get further distance to sink node will be more energy consuming and tend to paralyze in an early phase, which will affect the performance of the entire network.
4. The entire network topology is limited to two hops which could not meet the demand of large-scale network.

From the above, the problems of LEACH focus on how to construct the cluster evenly distributed and make the node with larger residual energy become the head, and how to reduce the communicational cost between clusters. 

Some researchers proposed that the energy-LEACH [9] algorithm use is initial energy of node as a parameter. Nodes with the higher energy are more likely to be cluster heads. The disadvantage of the algorithm is that it has an uneven distribution of cluster which may lead to high communication cost. In BSCA protocol [10], nodes nearer to the sink node are more likely to become cluster heads. However, this protocol has its shortcoming that cluster head nodes are too concentrated on the vicinity of the sink node, and nodes which are far away from sink node have a larger communication overhead. There is another protocol named LEACH-C (LEACH-centralized) protocol [11], which chooses its cluster head by using clustering algorithm according to node energy and location. Therefore, the chosen head has enough energy to communicate and has relative dominance in communication distance. However, in every process of clustering, each node has to communicate with sink node, which will raise the cost and increase the time delay.

For interclusters communication, there is the CMRA [12] algorithm. It gradually establishes the minimum energy path tree between the cluster head nodes and sink node. Nevertheless, it does not take the distance between cluster heads into account when building the minimum energy path tree. Another algorithm related to LEACH is the LEACH-MF [13] that pays attention to make new hierarchical relations within the heads. Its disadvantage lies in that efficiency of LEACH-MF will decrease rapidly because of the two-hop routing if the network size is increased.

How to determine the route between head cluster and sink node? Some classical physics models can be mapped to wireless sensor network. In [14], Barraquand et al. abstracted the model of wireless sensor network as electric charge in the electric field and got the conditions of optimal distribution for wireless sensor network node distribution. This algorithm requires that nodes should have mobility which is energy consuming for the mobile devices of nodes. Network nodes are divided into different depth by means of broadcasting in paper [15]. It quoted to the concept of the potential field in physics to simulate wireless sensor network into a virtual potential field, constructing the routing structure based on the potential field and completing the data transfer.

This paper aims to construct a virtual potential field [16], where each node will have field strength and be divided into different equipotential fields according to the field strength. Every node has the same height in the same equipotential field. Then field strength and the height of the potential field are integrated to get the potential energy of the node. According to the potential energy difference of the adjacent potential field between two nodes, data flow will be generated from the high potential node to the low potential node. It could establish a routing tree of wireless sensor network based on the data flow and complete the transmission of data.

## 3. Network Model and Problem Description

### 3.1. Network Model

Numerous nodes are randomly deployed within the *M*∗*M* area, which have the following characteristics.

(1) All nodes are isomorphic.
(2) The deployment location of nodes is already known and the nodes could not move after deployment. All nodes do not require manual maintenance. The energy of nodes is nonrenewable.
(3) All nodes have the ability of sensing, wireless communication, and data fusion. The communication power can be adjusted according to communication distance.
(4) There is only one sink node within the region of the *M*∗*M* and all the nodes can communicate with the sink node. The energy of sink node is considered as positive infinity.
(5) The nodes transfer data by means of wireless communication which was referred to in article [17] with the threshold of *d*_0_. Here is the formula of *d*_0_:
  (1) $$\begin{array}{c} d_{0}=\sqrt{\frac{E_{fi}}{E_{mp}}}. \end{array}$$

*E*_*fi*_ stands for the energy consumed by sending 1 bit data in free space model. *E*_*mp*_ stands for the energy consumed by sending 1 bit data with multipath fading model.

### 3.2. Problem Description

The question mainly focuses on the optimization of clusters and route between cluster head nodes based on the overview of LEACH algorithm and its improved algorithm for wireless sensor network.

The problem of constructing cluster can be considered into two aspects. The first one is how to optimize the number of cluster head nodes while cluster generating. If there are too much cluster heads, the size of the intercluster routing tree will increase. As a result of that, data transmission needs more hops and the load of the nodes close to the sink node will increase either, which would lead to the premature death of these nodes. On the other hand, if there are less cluster heads, the size of the routing tree will decrease and the average communication distance of prehop increase, which leads to higher energy consumption of the cluster head node and is not conducive to extend the network lifetime.

The second problem comes from that the nodes in wireless sensor network are deployed randomly. This will probably cause too many members in some clusters and overlarge radius of them. Thus the communication cost will increase, which makes against improving the network's energy consumption.

LEACH-PF is a multihop routing tree algorithm based on energy potential field. The network is divided into a number of small areas. Each node deployed in any area consists of a cluster. The communication radius of cluster must be limited to the scope of “*d*_0_.” The intercluster routing tree is set up according to the data flow path, which is derived from potential energy difference between the nodes based on the potential field model so as to preferably balance the communication distance and the average number of hop.

The building process of the LEACH-PF algorithm for each round is as follows.

Network area distribution → cluster head node distribution → determination of cluster head node → routing tree generation → data communication.

## 4. The Partition Cluster Multihop Routing Tree

### 4.1. Virtual Energy Potential Field Model

This paper proposed a virtual energy potential field model. All the nodes are in a potential field excited by the sink node. The field direction of each node points to the sink node in the network area. The field strength is related with node's current residual energy. The entire region is defined as the virtual energy potential field and is divided into different potential field is according to the size of node potential. There is a difference between the high potential node and the low potential node. This potential energy difference prompts the trends from high potential node to the low potential one. Mapped to wireless sensor network, there is a trend that makes the high potential node forward data transmission to the low potential node. It could establish a connection between two nodes based on the trend of the data flow so as to constitute over a multihop routing tree after establishing a connection between all nodes.

The node potential energy height is calculated according to the location coordinate and the current residual energy of the node, and the height of the potential field. *E*_*fi*_ indicates the field strength of *i* node. *E*_*fi*_ is calculated as follows:

(2) $$\begin{array}{c} E_{fi}=-\frac{E_{c}}{D_{i}^{2}}, \end{array}$$

where *E*_*c*_ is the residual energy of the node and *D*_*i*_ is the distance between node *i* and sink node. *H*_*i*_ is the equipotential height of the nodes in the potential field. *H*_*i*_ is related to the network area size *L* and the sequence of all cluster head node's field strength:

(3) $$\begin{array}{c} H_{i}=f\left(E_{fi},L,N_{c},d_{0}\right), \end{array}$$

where *N*_*c*_ is the sequence number in a sequence which sorts by the size of *E*_*fi*_. *L* is the diameter of the network. *P*(*i*) is the potential energy:

(4) $$\begin{array}{c} P\left(i\right)=\lambda \cdot E_{fi}\cdot H_{i}+k, \end{array}$$

where *λ*, *k* are parameters. The potential size of each cluster head node can be obtained by formula (4). There would be a potential difference between the cluster head nodes because the potential energy of cluster head nodes is different. The node with high potential energy will transmit data to the node with low potential energy. The potential energy of the sink node is set to negative infinity. All data will eventually converge to the sink node. We could build a global routing whose root is sink node along with the node data transmission path to complete the task of collecting monitoring data in wireless sensor network.

This paper proposed partition multihop routing tree algorithm LEACH-PF on the basis of LEACH. The algorithm round is divided into three stages: clustering, constructing the routing tree, and data transmission.

### 4.2. Clustering Stage

In paper [18] the whole network area is divided into a number of square partitions, which makes the cluster distribution more uniform and to some extent reduce the energy consumption. However, the distribution of cluster area is based on theoretical value, causing the different size of areas. Thus, the farther distance between cluster head and clustering members will make the intercluster head nodes consume more energy. It suggested dividing the entire wireless sensor network into a number of square areas with each partition assigning with a cluster head. At the same time, it should be ensured that the distance between the cluster head and nodes cannot exceed the threshold *d*_0_, so that the communication consumption between the members of the cluster and the cluster nodes can be maintained at a low level. The methods of cluster constitution are as follows.

(1) When it is the first round according to the cluster head selection mechanism of LEACH, select the cluster head node randomly and the ordinary nodes join to the nearest cluster. The ordinary nodes transmit their own position coordinates and the ID of each node to cluster head with the first round of transmission data. Then cluster head transmits the position coordinates of the cluster members and their ID to sink node.
(2) The sink nodes divided the network into a number of square areas by calculating the size of network. The square's diagonal size is *d*_0_. The number of squares is calculated as
  (5) $$\begin{array}{c} N_{c}=\frac{\left(M*\sqrt{2}\right)}{d_{0}}. \end{array}$$
(3) According to the first round, the sink nodes decide which node belongs to which square area and broadcasts it to the entire area, so that each node would know which region itself belongs to. The delay of the broadcast process and the calculation process is negligible due to the high computation ability of the sink node and unlimited energy.
(4) Each square area will get a cluster head in each round after the first round. Cluster members send their own node ID and residual energy to the cluster head node. Cluster selects the maximum residual energy node as cluster head of the next round and then broadcasts it to cluster members that which is the next cluster head and sent it to the sink node through the energy potential routing tree.
(5) After the first round data transmission, nodes join to next cluster head and repeat step (4).

**Figure 1:**
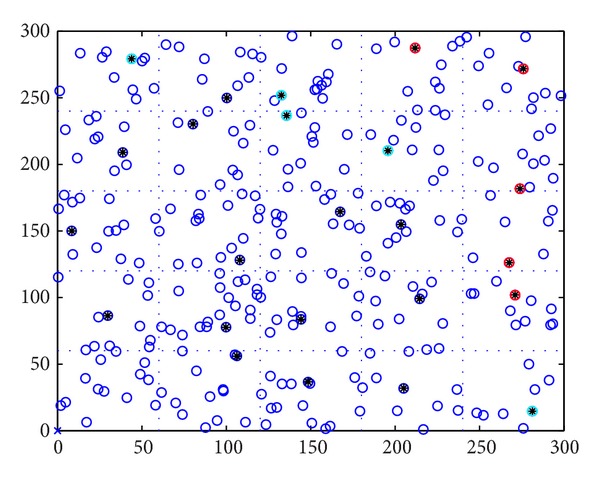
The division of network.

After many experiments, it turns out that this kind of area distribution makes the distribution of cluster heads more uniform compared with LEACH. The distance of cluster head broadcast and data sending of cluster members is less than *d*_0_. This algorithm is effective for balancing energy consumption and saving node energy.  Figure 1 is the division of network. Nodes were randomly distributed in the 300 m ∗ 300 m area. The whole network was divided into 25 subnetworks by calculating the size of network with formula (5). Each subnetwork has a cluster head node. The hollow circle points are ordinary nodes and the solid points are cluster head nodes.

### 4.3. The Intercluster Multihop Routing Based on Energy Potential

In Section 4.2 the cluster has been established. Field strength of each cluster head *E*_*fi*_ is calculated. All the nodes are divided into different potential fields according to *E*_*fi*_. Nodes in the same equipotential field have the same priority of data transmission. The equipotential height of the nodes in the potential field is defined as follows:

(6) $$\begin{array}{c} H\left(i\right)=\left\lfloor \frac{N}{S}\right\rfloor . \end{array}$$

*S* is the number of potential fields. *N* is the Sequence of sort *E*_*fi*_ in all potential fields. *N* is calculated as follows (Algorithm 1).

After these processes, the cluster head nodes have been divided into different potential field, and their potential energy has been already calculated.

Building the routing tree is the next step. The potential difference between node *i* and node *j* could be calculated in accordance with formula (7):

(7) $$\begin{array}{c} \Delta P\left(i,j\right)=\left|\frac{P\left(i\right)-P\left(j\right)}{d}\right|, \end{array}$$

where *d* is the distance between the node *i* and node *j*. *R*(*i*) is a set of candidates next hop of node *i*. All the nodes in the next adjacent equipotential field of node *i* were joined to *R*(*i*). Node *i* selects the optimal next hop node *j* from *R*(*i*):

(8) $$\begin{array}{c} \text{next}\left(i\right)=\left\{j\;|\;\max\left(\Delta P\left(i,j\right)\right),\;j\in R\left(i\right)\right\}. \end{array}$$

**Figure 2:**
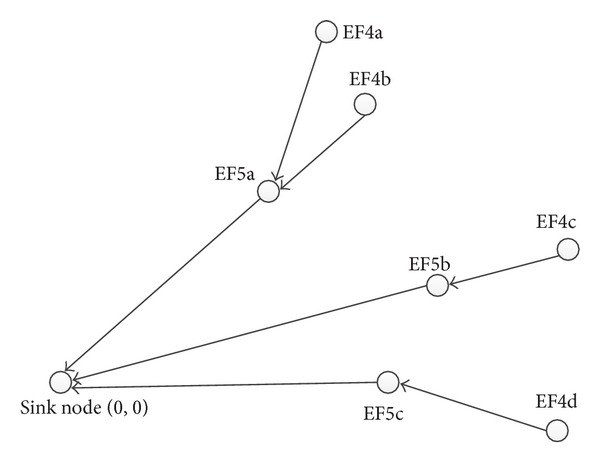
The diagram of establishing a routing tree.

Node *i* finds the next hop node and establishes a connection between the two nodes in accordance with (8). The nodes in other potentials could find their next hop node in the same way. The last equipotential field nodes establish a connection with the sink node directly.  Figure 2 is a diagram to establish a routing tree.

In Figure 2, the node marked sink node is the root and the node marked EF*xy* means that this node is the number *y* node in the *x* equipotential field. It can be seen from the figure that the node from the 4th equipotential field tends to find next hop node in the 5th equipotential field. EF4a will choose EF5a as its next hop, because EF5a is the nearest to it geographically among all the nodes of the 5th equipotential field. Similarly, the EF4b, EF4c, and EF4d could also find their next hop node separately.

A routing tree comes into being after all the cluster head nodes find their next hop nodes. Sensor nodes gather sensor environment data and send the data to their own cluster head nodes. Cluster head node combines the data of other ordinary nodes, sends the data to sink node through the multihop routing tree, and performs the task of monitoring the wireless sensor network environment.

## 5. Simulation Analysis

We compared the efficiency of LEACH-PF and other improved LEACH algorithms. The numerous nodes are randomly deployed in *M*∗*M* network area. The numbers of rounds in the experiments are regarded as a measure to evaluate the lifetime of network. The parameters of the simulation experiments are shown in Table 1.

### 5.1. Experimental Results

A comparison is made between LEACH-PF algorithm and LEACH, LEACH-MF, and CMRA algorithms under the same simulation parameters except the nodes number. The average energy consumptions of each round for different algorithms are shown in Table 2, which are calculated before the first dead node occurs.

As can be seen from Table 2, in the same network area, there is only a little difference between the four protocols when the number of nodes is small. With the increasing of number of the deployed nodes, the average energy consumption of LEACH-PF isthe smallest than other protocols. That means that LEACH-PF consumes less energy than other protocols.

In the next experiment compared with CMRA and LEACH-MF, nodes are distributed in the area of 300 m ∗ 300 m with the initial energy setting at 0.5. The rounds when the first node is dead and half of the nodes are dead are counted separately, as well as the average energy consumption of nodes in each round. The experimental results are as follows.

**Figure 3:**
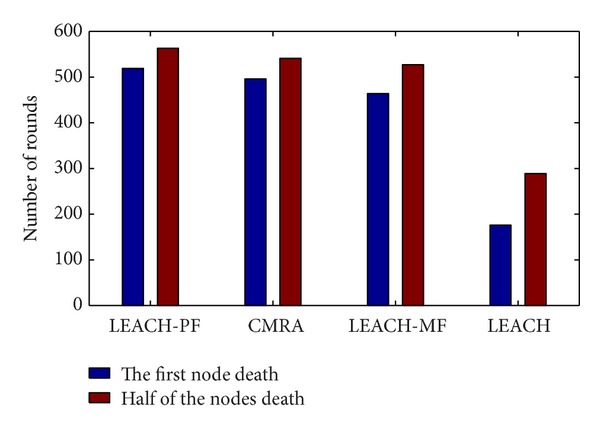
The comparison of node death round.

In Figure 3, the blue column represents the rounds of the first dead node while red column represents the rounds of the death of half of the nodes. As can be seen from Figure 3, from the highest to the lowest, the rounds of the first dead node sequence are LEACH-PF (519R), CMRA (496R), LEACH-MF: (464R), and LEACH (176R). By the same way, the rounds of the death of half of the nodes sequence are LEACH-PF (563R), CMRA (541R), LEACH-MF: (527R), and LEACH (289R). It shows that LEACH-PF has the longest lifetime in either case.

**Figure 4:**
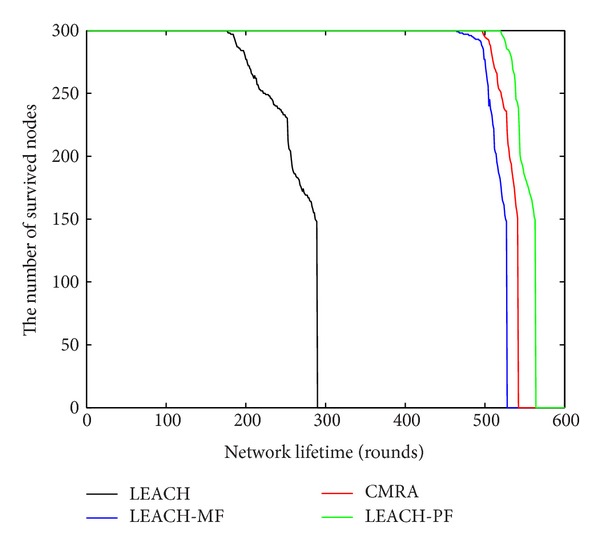
Four algorithms increase with the number of rounds remaining nodes.

In Figure 4 the green curve is the number of survived nodes with the increase of rounds for LEACH-PF. The other curves indicate the same for different algorithms separately, such as red curve for CMRA, blue curve for LEACH-MF, black curve for LEACH. Figure 4 shows that LEACH-PF could survival more nodes than the other three algorithms.

**Figure 5:**
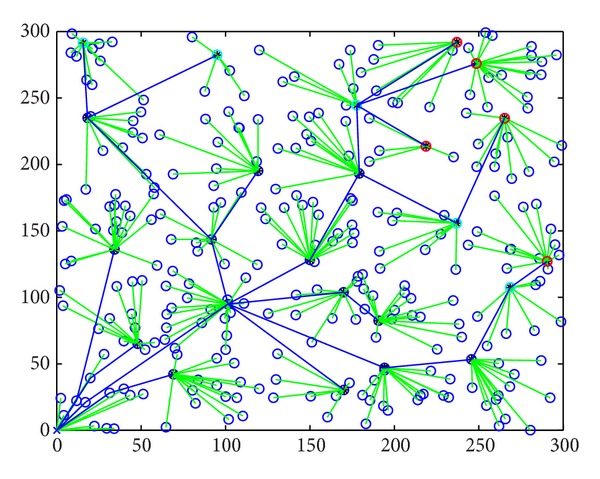
The topology for LEACH-PF.

Figure 5 shows the topology of the LEACH-PF algorithm. The size of network area is also 300 m ∗ 300 m. The network should be divided into 5 ∗ 5 small areas according to LEACH-PF. Every area is assigned a cluster head node. The cluster head constitutes a multihop routing tree successfully.

## 6. Conclusion

On the basis of hierarchical routing protocol, this paper proposed an improved clustering and cluster head selection mechanism with distribution position and the remaining energy of nodes. We use the potential difference between the different equipotential fields to construct the intercluster communication routing tree. The experimental results show that LEACH-PF postponed the rounds of first node death and improved the monitoring time of network to environment and extended the network lifetime.

 As considered in this paper, the area division is conducted by means of the wireless communication threshold *d*_0_. When extended to large-scale network, this algorithm may divide network into an excessive number of subnetworks. This will increase the load of the nodes close to the sink node and cause energy holes significantly. How to balance load and the data forwarding of nodes will be the focus of future research.

## Figures and Tables

**Algorithm 1:**
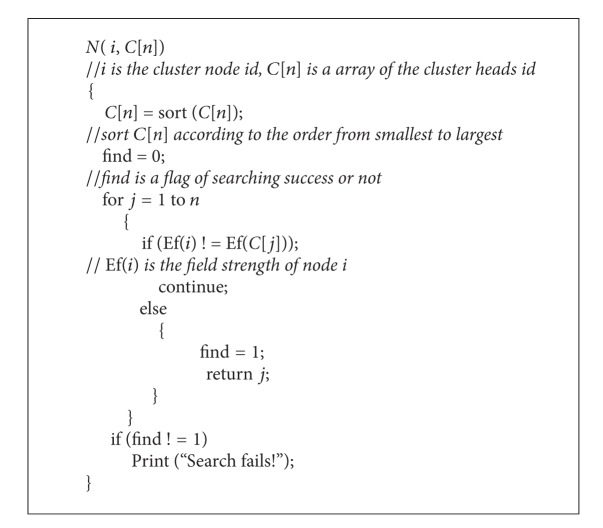


**Table 1:** Simulation parameters.

Network size	300 m∗300 m
Sink node locations	(0, 0)
Data packet size	4000 bit
Control packet size	100 bit
Initial energy of node	0.5 J
*E*_*tx*_	5∗10^−9^ J/bit
*E*_*rx*_	5∗10^−9^ J/bit
*E*_*fs*_	10^−12^ J/bit∗m^2^
*E*_*mp*_	0.0013∗10^−12^ J/bit∗m^4^
EDA	5∗10^−9^ J/bit/signal

**Table 2:** The average energy consumption when the first node is dead.

Number of nodes	LEACH	LEACH-MF	CMRA	LEACH-PF
100	0.193 J	0.191 J	0.183 J	0.180 J
300	0.418 J	0.274 J	0.252 J	0.240 J
500	1.066 J	0.449 J	0.416 J	0.395 J

## References

[1] Jianmin S (2006). *Wireless Sensor Network*.

[2] Ammari HM, Gomes N, Grosky WI (2012). Review of applications of wireless sensor networks. *Wireless Sensor Networks: Current Status and Future Trends*.

[3] Durisic MP, Tafa Z, Dimic G A survey of military applications of wireless sensor networks.

[4] Shen B, Zhang S-Y, Zhong Y-P (2006). Cluster-based routing protocols for wireless sensor networks. *Journal of Software*.

[5] Viani F, Salucci M, Rocca P A multi-sensor wsn backbone for museum monitoring and surveillance.

[6] Kumar CKS, Sukumar R, Nageswari M (2013). Sensors lifetime enhancement techniques in wireless sensor networks—a critical review. *International Journal of Computer Science and Information Technology & Security*.

[7] Waware S, Sarwade DN, Gangurde P (2012). A review of power efficient hierarchical routing protocols in wireless sensor networks. *International Journal of Engineering Research and Applications*.

[8] Heinzelman W (2000). *Application-Specific Protocol Architectures for Wireless Networks*.

[9] Xiangning F, Yulin S Improvement on LEACH protocol of wireless sensor network.

[10] Zytoune Q, Fakhri Y, Aboutajdine D (2009). A balanced cost cluster-heads selection algorithrn for wireless sensor networks. *International Journal of Computer Science*.

[11] Heinzelman WB, Chandrakasan AP, Balakrishnan H (2002). An application-specific protocol architecture for wireless microsensor networks. *IEEE Transactions on Wireless Communications*.

[12] Haoran L, Yan F, Xiaochen H, Jingjing D, Hongliang L, Weihong B Research of inter-cluster multihop routing algorithm for wireless sensor networks.

[13] Yan J-F, Liu Y-L Improved LEACH routing protocol for large scale wireless sensor networks routing.

[14] Barraquand J, Langlois B, Latombe J-C (1992). Numerical potential field techniques for robot path planning. *IEEE Transactions on Systems, Man and Cybernetics*.

[15] Janabi Sharifi F, Vinke D Integration of the artificial potential field approach with simulated annealing for robot path planning.

[16] Caselli S, Reggiani M, Rocchi R Heuristic methods for randomized path planning in potential fields.

[17] Heinzelman WB, Chandrakasan AP, Balakrishnan H (2002). An application-specific protocol architecture for wireless microsensor networks. *IEEE Transactions on Wireless Communications*.

[18] Duan CQ, Sun JJ, Zhou D (2010). Energy-efficient region-partitioned clustering multihop routing algorithm in WSN. *Computer Engineering*.

